# Observational study of the microbiome of perforated abomasal ulcers in unweaned beef calves in Canada

**DOI:** 10.1007/s11259-026-11526-4

**Published:** 2026-09-18

**Authors:** Renée M. Petri, Sara Ricci, Murray Jelinski, Alexandra Hund

**Affiliations:** 1 Sherbrooke Research and Development Centre, Agriculture and Agri-Food Canada, Sherbrooke, Canada; 2 Centre for Animal Nutrition and Welfare, Clinical Department for Farm Animals and Food System Science, University of Veterinary Medicine Vienna, Vienna, Austria; 3 Western College of Veterinary Medicine, University of Saskatchewan, Saskatoon, Canada; 4 Agricultural Center for Cattle Production, Grassland Management, Dairy Food, Wildlife and Fisheries of Baden-Wuerttemberg (LAZBW), Aulendorf, Germany

**Keywords:** Perforating abomasal ulcers, Metagenomics, 16S, Beef, Antibiotics, Resistance genes, Postmortem

## Abstract

In western Canada, perforating abomasal ulcers (AU) are generally diagnosed postmortem in beef calves up to 2 months of age. Certain microbes have been associated with AU, but the evidence is circumstantial. Thus, analyzing the abomasal microbiome in calves with and without AU may provide insight into the etiology of the disease. Using 16S rRNA gene sequencing, abomasal tissues from Western Canadian beef calves, with (*n* = 27) and without AU (*n* = 17), were analyzed for microbial diversity. No significant differences were seen between microbiomes of AU and CON calves despite numerical differences in abundance. Therefore, a subsample of 18 tissues (12 AU, 6 CON) were analyzed using untargeted metagenomic sequencing for determination of phylogeny, and the presence of antimicrobial resistance genes (ARGs). *Staphylococcaceae* (1.5%), *Campylobacteraceae* (1.4%) and *Enterobacteriaceae* (1.4%) were seen across all samples. Differential abundance analysis revealed *Streptomyces* spp. REN17 to be less abundant in AU calves, suggesting a potential association between *Streptomyces* spp. and calf gut health. An ARG associated with polymyxin resistance was found to be differentially abundant in calves with a history of antimicrobial therapy. Although differences were observed in the predicted metabolic functions among groups, none reached statistical significance. In our study, no consistent microbial signature associated with AU was identified, despite the differential abundance of individual taxon, nor did prior antimicrobial therapy associate to the growth of specific microbiota. To better understand the potential role of the abomasal microbiota in AU etiology and the impact of early life interventions in beef calves, a larger sample size is needed.

**Supplementary Information:**

The online version contains supplementary material available at https://doi.org/10.1007/s11259-026-11526-4.

## Introduction

The role of abomasal ulcers (AU) in adversely affecting health and production in cattle is not fully understood. Across the various cattle production systems, veal calves are the most severely affected by AU (Hund and Wittek 2018). Perforating AU, the most harmful type, have been found to affect 0.11 to 0.54% of European veal calves in group housing (Bähler et al. 2012; Pardon et al. 2012). Compared to veal calves, less is known about the prevalence of AU in range beef calves, and most perforating AU are diagnosed postmortem (Jelinski et al. 1996a). In western Canada, perforating AU generally occur in calves less than two to three months of age, which coincides with the preruminant and transitional stages of forestomach development and the accompanying microbiome changes (Leek 1993). Waldner et al. (2010) found that abomasal ulcers and abomasal perforation accounted for 3.8% and 9.9% of diagnoses at the time of postmortem examination in calves 3 days to 3 months of age in western Canadian beef herds, indicating the significant economic impact of AU on cattle production.

Despite past research into the aetiology of perforating AU, a consensus on the causality has not yet been reached. However, it is well accepted that the aetiology of AU is likely multifactorial including stress and environmental factors (Berends et al. 2012). Beef calves are generally found to have a singular fatal perforating AU, typically found along the greater curvature of the fundus (Jelinski et al. 1996b). As these ulcers lead to the loss of the calf, understanding the etiology of unweaned beef calf perforating AU could have an impact on calf management strategies resulting in improved animal health.

In addition to nutritional effects, some studies have pointed towards the role of microorganisms in the pathogenesis of AU (Jelinski et al. 1995; Hund et al. 2015). In early life, the microbiome development of calves is under continual change (Malmuthuge and Guan 2017), as is physiological development of the foregut. The continuous transition prior to having a stable commensal population may make the abomasum more susceptible to disturbances such as AU (Rey et al. 2014). Previous research has implicated a number of pathogens, such as *Clostridium perfringens*, or *Campylobacter* spp. as being associated with AU, but evidence is circumstantial and the true role of the pathogens is unknown (Roeder et al. 1987; Jelinski et al. 1995; Valgaeren et al. 2013). However, with the advancement of culture-independent techniques, the capacity to identify microbes, not only phylogenetically but also functionally, has improved the resolution of knowledge for more fastidious gut microbes in complex ecosystems in both healthy and diseased states (Chistoserdova 2009).

An additional concern with perforating AU is that beef calves may have a history of having received antimicrobials at some point in their life, which may exacerbate microbial dysbiosis and increase antimicrobial-resistant organisms, as has been shown in humans (Matzaras et al. 2022; Kesavelu and Jog 2023). Thus, analyzing the abomasal microbiome for both taxonomy and the presence of ARG may provide some insight into potential pathobionts as well as the impact of antibiotic usage on antimicrobial resistance. The aim of this research was to assess the microbial diversity, composition, and functional potential, as well as the prevalence of antimicrobial resistance genes from western Canadian beef calves with and without AU.

## Materials and methods

This study was approved by the University of Saskatchewan Animal Care Committee (AUP#: 003CatA2020).

Volunteer clinic enrollment in the project was done through contact with diagnostic laboratories and practicing veterinarians. Practitioners were sent a ready-to-use sample kit consisting of collection cups, RNAlater (pH 5.2; Qiagen, Netherlands), sample collection instructions, and a history data sheet that included questions regarding antimicrobial treatments.

Sample collection instructions stated that the best efforts should be made to avoid cross-contamination when obtaining abomasal tissues. For each sample, a pie-shaped piece of tissue was cut from the edge of the perforation. The mucosal surface was carefully rinsed with saline solution, and the tissue samples placed in RNAlater solution. The ratio between sample volume and RNAlater was approximately 1:5. Samples were gently mixed and allowed to stand at room temperature for 30 min to 6 h before being stored in the freezer (-20 °C).

In total, samples from 45 animals were collected from one diagnostic laboratory and 12 veterinary clinics. The AU group consisted of unweaned calves less than four months of age that had died of a perforating AU (*n* = 30). The control group consisted of calves that showed no pathological changes in the abomasum at necropsy (CON; *n* = 15). In eight of these CON cases, the cause of death could not be determined on necropsy. Four calves died from mechanical GI obstruction (abomasal volvulus, intestinal volvulus, hairball obstructing the jejunum, and small intestinal strangulation through a diaphragmatic hernia) and one calf showed signs of hemorrhagic enteritis. Two calves died from presumed septicemia, one from meningitis and one was euthanised due to a necrotizing ischemia caused by salmonellosis. While the control group was heterogeneous, the animals were included as they did not exhibit abomasal ulcers, and while unrelated causes of death may contribute to additional biological variation, it was expected that this could reduce not enhance ulcer-associated microbial differences, therefore providing more conservative findings. When the groups were divided by age based on 30 days of age or less and 31 days to 3 months of age, a total of 43 samples could be grouped based on the provided data. Based on sex, 13 females and 8 males were identified from the total sample number, and all samples were collected between 1 and 24 h postmortem with the exception of one sample collected at 64 h postmortem. Seventeen samples were from animals that had records of previous parenteral antibiotic treatment. In 12 of those, the time since the last treatment was recorded (0–45 days, mean 8 days, median 1 day, SD 14.8). Breeds were identified as belonging to Charolais, Simmental, Angus, Limousin, Herford, or a commercial cross.

### Statistical analysis of the background information

The data were assessed for missing variables under the groupings of sex (male/female), age (over 30 d of age (> 30) vs. 30 d of age or less (≤ 30)), and antibiotic use (AB: antibiotic usage or NoAB: no antibiotic usage) when samples were separated into AU or CON (having no AU). Chi-Square tests were performed using SAS (9.4) for the association between tissue type (AU or CON) and each of the grouping separately (AB: *n* = 41; sex: *n* = 21; age: *n* = 39). Additionally, a logistic analysis of the combination of age and antibiotics was performed on 39 out of the original 45 observations with tissue type as the independent variable.

### DNA extraction and 16S rRNA gene sequencing

DNA extraction was performed with all 45 samples using the PowerSoil^®^ DNA Isolation Kit (QIAGEN^®^, Venlo, Netherlands) according to the manufacturer’s instructions. Then, the DNA concentration was determined using Nanodrop and 16S rRNA gene sequencing was performed using the Illumina MiSeq and the V2 500 cycle kit using paired-end technology (Illumina, San Diego, USA). The highly variable region V4 of the bacterial 16S rRNA gene was amplified (515F: GTGYCAGCMGCCGCGGTAA and 806R: GGACTACHVGGGTWTCTAAT). All sequencing was performed by Genome Québec (Montréal, QC, Canada) according to manufacturers’ procedures.

### Bioinformatic analysis of 16S rRNA gene sequencing data

Demultiplexing, trimming of adaptors, merging of reads, and quality analysis were performed with all 45 samples using QIIME 2 (v. 2023.5) (Bolyen et al. 2019). Merged sequences were filtered for quality (PHRED score > 30) before denoising with Deblur (Amir et al. 2017). Reads were trimmed at 251 nucleotides. Two samples did not pass the quality filtering and denoising resulting in a minimum number of reads per samples of 7000 (*n* = 43 samples remaining). The resulting sequences were further filtered to exclude mitochondrial and chloroplast contamination. Taxonomy was assigned with a Naive Bayes classifier trained for the specific 16S rRNA gene target regions against the database SILVA version 138 with 99% identity OTU reference (Quast et al. 2013). The filtered amplicon sequence variants (ASVs) tables were used to calculate Chao1 richness, Simpson diversity, and Shannon index (Kaehler et al. 2019) using phyloseq (R Core Team 2022; v1. 34.0; McMurdie and Holmes 2013).

### Statistical analysis of 16S rRNA gene sequencing and subsample criteria

Data was then divided into groups for the descriptive data analysis (*n* = 43). These samples were tested for normality using each response variable and then analyzed using the Wilcoxon non-parametric analysis based on the descriptive variables age (over 30 d of age (> 30) vs. under 30 d of age (≤ 30), antibiotic treatment (AB: antibiotic usage or NoAB: no antibiotic usage), sex (male or female) using the tissue type (AU vs. CON) as a factor (SAS v9.4). Diversity parameters were not affected by any of the descriptive variables in the background information, but variation was seen in the relative abundance of certain taxa. The level of significance for all statistical analyses was *p* < 0.05.

### Metagenomic sequencing and biostatistical analysis

For metagenomic sequencing, a selection of 18 subsamples (12 AU, 6 CON) containing samples with complete information on tissue type, age and antibiotic treatment were chosen. These samples were then selected from in order to have balanced proportions for analysis based on age, and antibiotic use (Supplemental Table 1). Calves were divided between the age groups into ≤ 30 (*n* = 7) and > 31 days of age (*n* = 11). Of these 18 samples, 7 had recorded treatment with antibiotics in the days prior to death with the exception of one calf treated 30 days prior to death and the remaining 11 were recorded as not treated (NoAB). All 18 samples were sent to Genome Quebec for untargeted metagenomic sequencing using Illumina NovaSeq S4 PE150 at a depth of 35 M reads according to manufacturer’s instructions.

The sequences were filtered and trimmed for quality using fastp (v0.22.0; Chen et al. 2018), discarding reads with a mean Phred quality score below 15 within a sliding window of 4 bp or below 100 bp in length (Holman et al. 2023). The reads were mapped against the bovine and PhiX genomes using Bowtie2 (v2.2.5; Langmead and Salzberg 2012), and the sequences that were not mapped were extracted using SAMtools (v1.6; Danecek et al. 2021). The resulting files were then sorted and converted to fastq format using BEDtools (v2.30.0; Quinlan and Hall 2010). The reads obtained were used for downstream analyses. Taxonomy was assigned using kaiju (v1.8; Menzel et al. 2016), with the NCBI BLAST non-redundant protein database for archaea, bacteria, and viruses, and additionally including fungi and microbial eukaryotes. Taxonomic summaries were generated for each individual sample at the phylum, family, genus and species levels, including the unassigned reads. Summaries were merged using R package tidyr (R Core Team 2022; Wickham et al. 2022). Metabolic prediction was performed on the unassembled reads using HUMAnN 3.0 (v3.6, chocophlan database v201901_v31) using log transformation (Sheng et al. 2024). Relative abundances of the pathways were calculated and used as input for statistics. MEGAHIT (v1.2.9; Li et al. 2015) was used to assemble the reads over all the dataset and per each individual sample. The minimum contig length allowed was 1000 bp. The generated assembly for the whole dataset consisted of 11,247 contigs (total 26,289,522 bp, min 1000 bp, max 32,404 bp, avg 2,337 bp, N50 2,671 bp). The resulting assemblies were used as input for NCBI Antimicrobial Resistance Gene Finder Plus (AMRFinderPlus; v3.10.45; Feldgarden et al. 2021) in order to detect the presence of classes of genes involved in virulence and stress-response in addition to resistance. Package MaAsLin2 was used to test the differential abundances of taxa between the control individuals and the ones with AU at the level of family, genus and species (Mallick et al. 2021). The mixed model was run with antibiotic treatment and presence/absence of ulcer as the fixed effects, while age was included as random effect. The dataset was normalized using centered log-ratio transformation and the default Benjamini-Hochberg post-hoc correction was applied (Ricci et al. 2024). The contigs created with MEGAHIT were used to build an index with Bowtie2. The reads were then mapped to the reference index that was created using Bowtie2. The output sequences were converted and sorted using SAMtools. MetaBAT2 (v2.15) was used to bin contigs from the coassembly into metagenome-assembled genomes (MAGs), with an allowed minimum length of 2000 bp (Kang et al. 2019). CheckM (v1.2.2) was used to assess the quality of the resulting bins (Parks et al. 2015). The same steps were repeated for each individual sample, starting from the assembly built with MEGAHIT per each sample, in order to create as many MAGs as possible.

## Results

Descriptive statistical analysis of the samples showed that there were too many missing observations for sex (24 out of 45 = 53%) to be able to reliably relate it to the type of tissue (CON vs. AU). Nevertheless, an exploratory analysis of the remaining 21 available data points, revealed no association between sex and tissue type as ulcers were found in about 62% of both males and females (CON or AU, *p* = 0.96; Table 1). For the separation of age into 30 days of age or less and 31 days to 3 months of age (43 out of 45 = 95.6%), Chi-Square analysis showed a trend towards a mild association between age and type of tissue (Table 2; *p* = 0.08). For analysis of the 41 out of 45 samples with an indication of the exposure to AB, no association was seen with type of tissue (Table 3; *p* = 0.27). When age and AB exposure were analyzed jointly, 39 of 45 samples could be categorized, resulting in a Chi-Square test of *p* = 0.06 (Table 4). Therefore, there was insufficient evidence that the ulcer distribution differed significantly between categories. Subsequent logistic analysis showed that the number of ulcers found in older calves was higher than any other category, with the majority being in calves that did not receive AB treatment.

**Table 1:** Chi-Square analysis of abomasum samples based on gender

	Control (CON)		Ulcer (AU)			
Parameter	(*n* = 15)	% of total samples	(*n* = 30)	% of total samples	Percent of Total Samples	*p*-value
Sex (Total)	8	38.1	13	61.9		0.9649
Male	3	14.3	5	23.8	38.1	
Female	5	23.8	8	38.1	61.9	

Frequency Missing = 24

**Table 2:** Chi-Square analysis of abomasum samples based on age

	Control (CON)		Ulcer (AU)			
Parameter	(*n* = 15)	% of total samples	(*n* = 30)	% of total samples	Percent of Total Samples	*p*-value
Age (Total)	15	34.9	28	65.1		0.0776
≤ 30 days of age	9	20.9	9	20.9	58.1	
> 30 days of age	6	14	19	44.2	41.9	

Frequency Missing = 2

**Table 3:** Chi-Square analysis of abomasum samples based on antibiotic exposure

	Control (CON)		Ulcer (AU)			
Parameter	(*n* = 15)	% of total samples	(*n* = 30)	% of total samples	Percent of Total Samples	*P*-value
Antibiotic exposure (Total)	13	31.7	28	68.3		0.2728
NoAB	6	14.6	18	43.9	58.5	
AB	7	17.1	10	24.4	41.5	

Frequency Missing = 4

**Table 4:** Chi-Square analysis of abomasum samples based on antibiotic exposure and age

	Control (CON)		Ulcer (AU)			
Parameter	(*n* = 13)	% of total samples	(*n* = 26)	% of total samples	Percent of Total Samples	*P*-value
Antibiotic Exposure x Age (*n* = 39)						
NoAB		33.3		66.7		0.0558
≤ 30 days of age	5	12.8	5	12.8	25.6	
> 30 days of age	1	2.6	13	33.3	35.9	
AB						
≤ 30 days of age	2	5.1	4	10.3	15.4	
> 30 days of age	5	12.8	4	10.3	23.1	

Frequency Missing = 6

### Metataxonomics

**Fig. 1:**
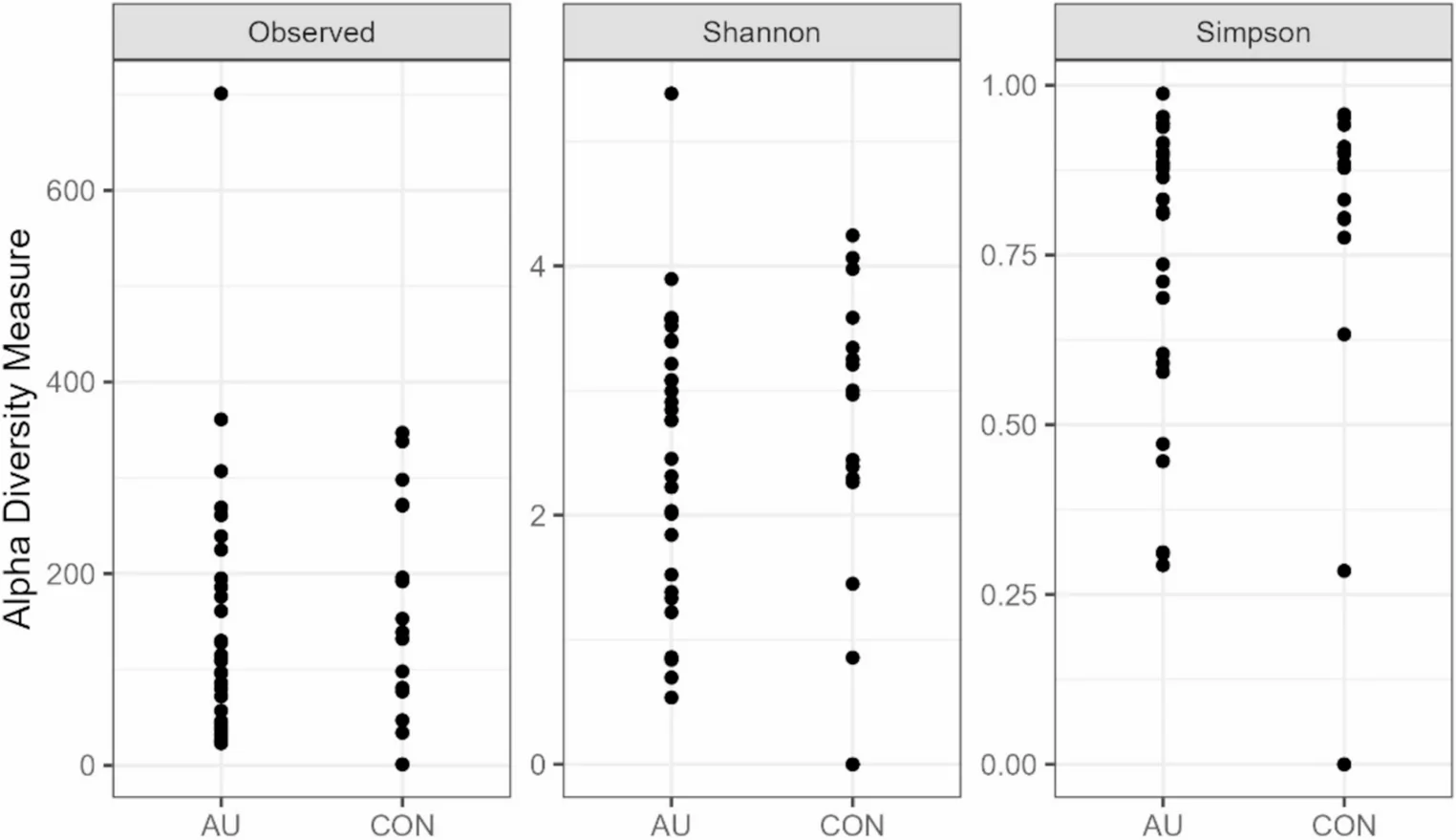
Distribution of calf abomasum samples between tissue types (abomasal ulcer: AU; no ulcer: CON) for various measures of alpha diversity (Observed: observed ASV; Shannon: Shannon index; Simpson: Simpson index)

To understand microbial sample variation, with regards to the presence or absence of AU, measures of alpha diversity were assessed after removal of the 2 samples that did not pass quality control (AU = 28, CON = 15; Fig. 1).

Shannon was normally distributed, whereas observed ASVs and Simpson index were not normally distributed. Therefore, the effect of tissue type, age, antibiotic use, and sex on diversity indices were evaluated using both a T-test for normally distributed measures and a non-parametric Wilcoxon Test for non-normally distributed measures (Supplementary Table 2). No significant associations were observed for any of the diversity measures in association with the type of tissue, the age of the animals, the previous exposure to antibiotics, nor the sex.

Relative abundance was also not normally distributed at the phylum level; therefore, analysis was completed with the non-parametric Wilcoxon test. Neither tissue type (AU vs. CON) nor antibiotic use (AB and NoAb) had an effect on the relative abundance of the 9 most commonly identified phyla (Table 5). Wilcoxon analysis was performed to examine the interaction between age and antibiotic usage based on the significant interaction noted for diversity parameters (Table 6). While *Bacteroidota* and *Firmicutes* shared an inverse pattern of abundance, only *Bacteroidota* was significantly impacted by the interaction between age and AB usage whereas *Firmicutes* showed a trend. *Campylobacterota* was also significantly increased with age, but also higher in animals given AB. At lower taxonomic levels, variation was attributed to unknown genera and therefore, data is not presented here.

**Table 5:** Wilcoxon p-values for impact of tissue type, age and prior antibiotic use on the relative abundance of phyla across samples from AU and CON calves

Phyla	Tissue Type	Age	Antibiotic Usage
*Actinobacteriota*	0.801	0.059	0.906
*Bacteroidota*	0.658	0.002	0.504
*Campylobacterota*	0.970	0.020	0.302
*Desulfobacterota*	0.872	0.63	0.924
*Euryarchaeota*	0.194	0.703	0.668
*Firmicutes*	1.0	0.054	0.136
*Fusobacteriota*	0.478	0.128	0.337
*Proteobacteria*	0.952	0.290	0.864
*Verrucomicrobiota*	0.263	0.262	0.749

**Table 6:** Wilcoxon values of the combination of age and antibiotic usage on the relative abundance of phyla across samples from AU and CON calves

		> 30 days of age		≤ 30 days of age		
		NoAB^1^ (*n* = 14)	AB (*n* = 10)	NoAB (*n* = 9)	AB (*n* = 6)	Wilcoxon Scores
*Bacteroidota*	Median	3.44 ^ab^	1.44 ^b^	9.15 ^a^	11.27 ^ab^	0.0347
	Score	17.07	14.40	28.11	24.00	
*Campilobacterota*	Median	0.00 ^b^	0.02 ^ab^	0.12 ^ab^	0.47 ^a^	0.0210
	Score	13.86	19.10	25.33	27.83	
*Firmicutes*	Median	82.50	95.29	61.25	76.52	0.0959
	Score	19.57	26.50	13.33	20.17	

^1^ NoAB: no antibiotics; AB: antibiotics; ^a,b^: different letters indicate statistically different values

### Metagenomics

Analysis of the metagenomic dataset only produced 9 MAGs that did not meet the quality filtering criteria. Given the limited completeness, number, coverage, and representation of these MAGS across samples, analysis with assembled reads was considered underpowered and was not pursued further.

**Fig. 2:**
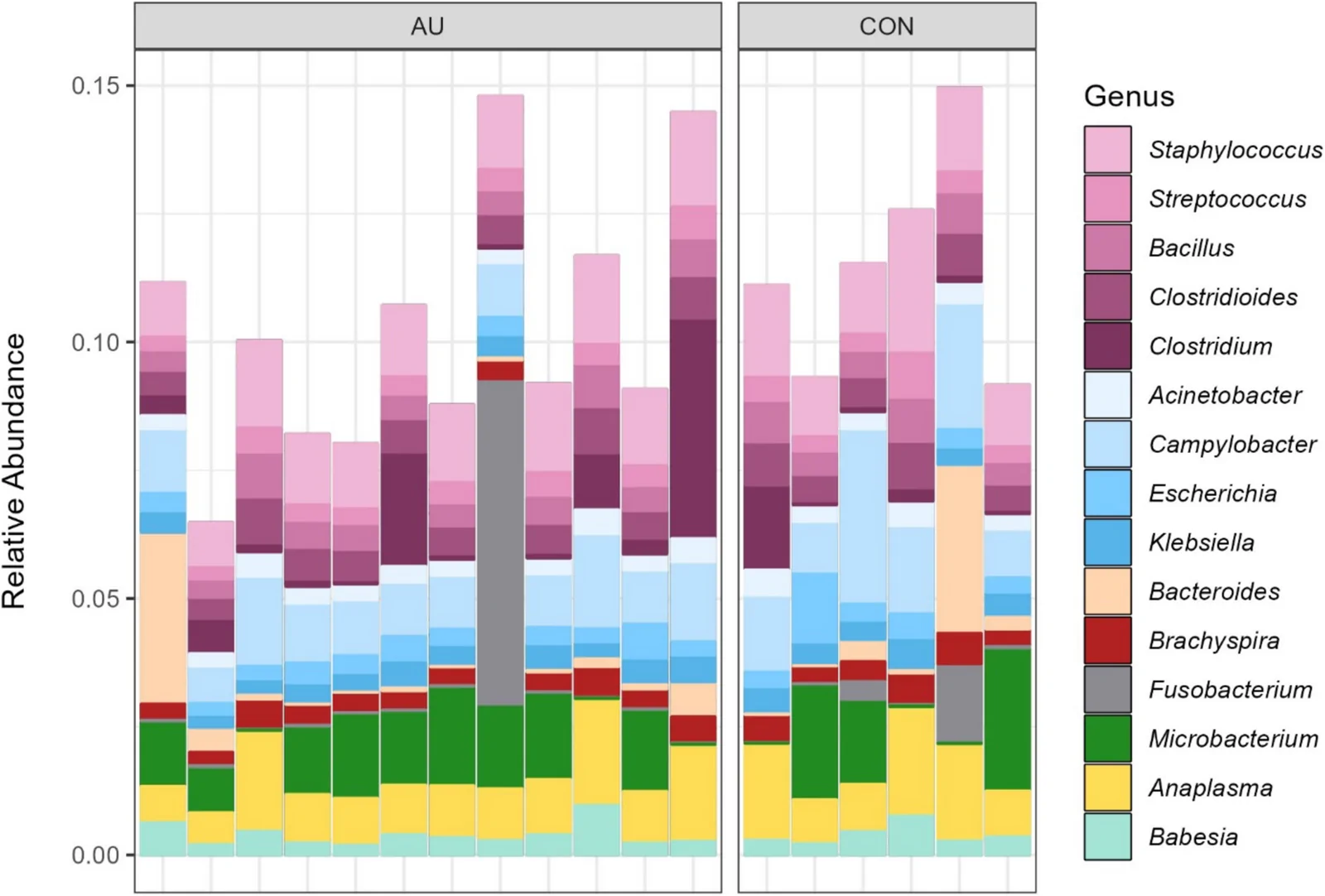
Relative abundance of the genus-level taxonomy of unassembled reads from the abomasum of unweaned calves with (AU) and without (CON) abomasal ulcers

The most prevalent taxonomy at the family level were *Staphylococcaceae* (1.5%), *Campylobacteraceae* (1.4%) and *Enterobacteriaceae* (1.4%) across all 18 samples, with *Staphylococcus aureus* (1.4%) as the most abundant species. Relative abundances of the most abundant genera are presented in Fig. 2.

**Fig. 3:**
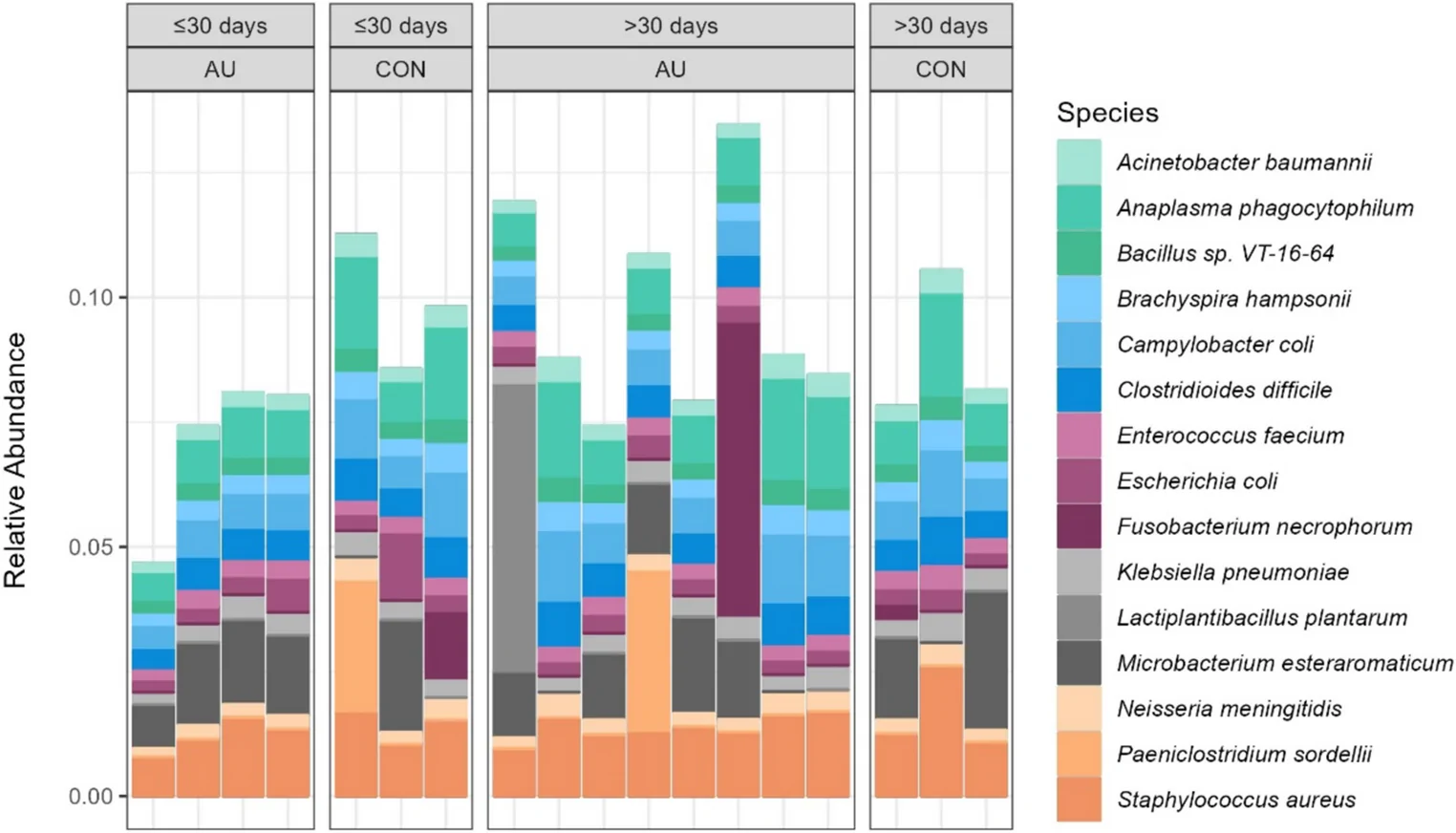
Relative abundance of identified species from unweaned calves 30 days of age or less (≤ 30) and over 30 days (> 30) of age between calves identified as having abomasal ulcer (AU) and without (CON)

Analysis for differential abundance was run with either fixed effects of tissue type, AB treatment or age. Relative abundances of most abundant identified species varied between samples more than between age groupings (Fig. 3), with low general diversity of species. Furthermore, separation of abundance of species between CON and AU samples showed no variation (*p* ≥ 0.05) within the most abundant identified species.

**Fig. 4:**
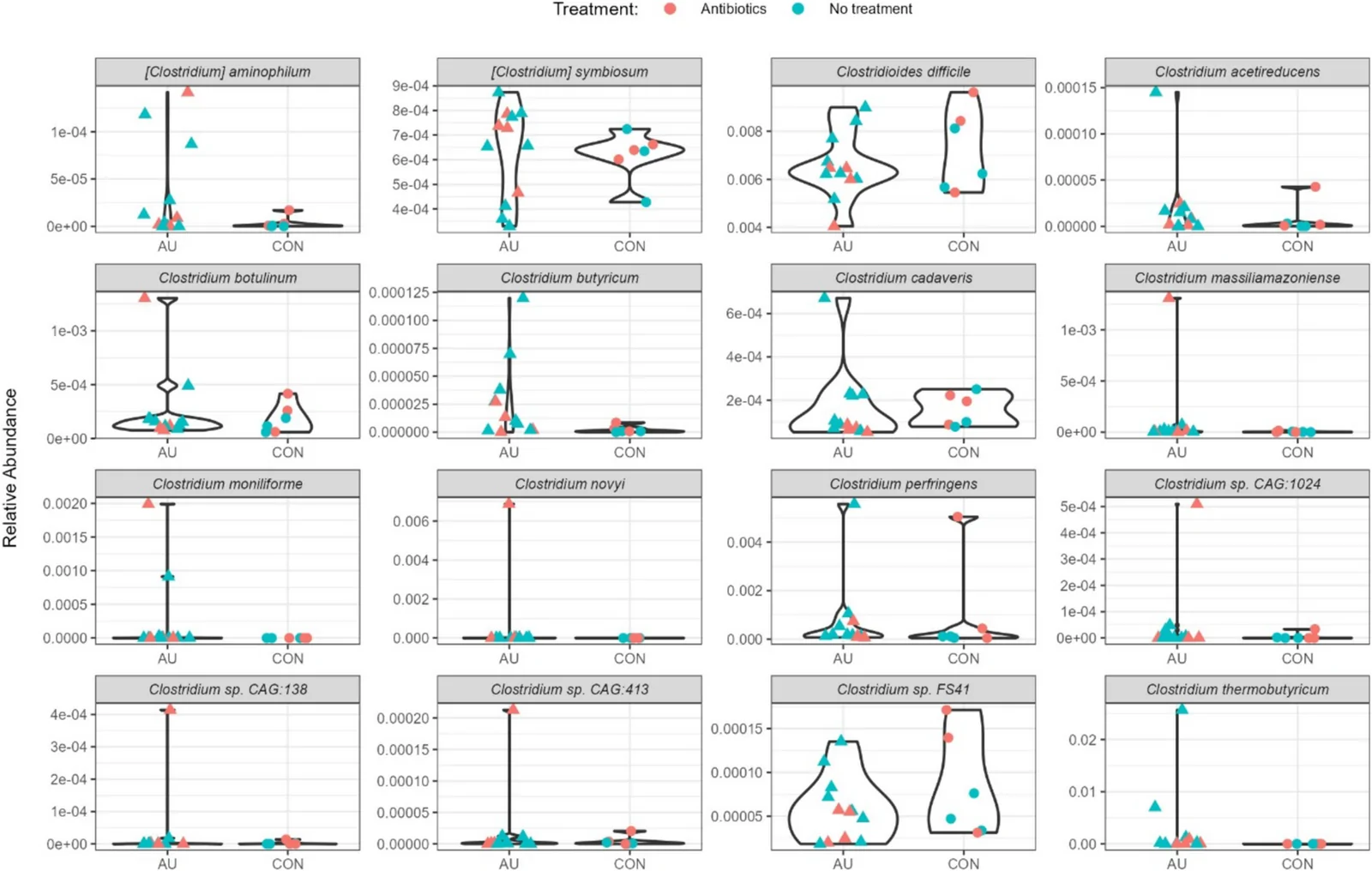
Differential abundance of identified sequences at the species-level taxonomy belonging to Genus *Closridium* from the abomasum of unweaned calves with (AU) and without (CON) abomasal ulcers (circle: CON, triangle: AU; red: Antibiotics (AB) vs. blue: NoAB)

MaAsLin2 analysis of differentially abundant species belonging to the genus *Clostridium* were done for CON and AU samples with and without AB treatment (Fig. 4) based on previous research implicating *Clostridium* in AU animals and a lack of *Campylobacter* in the current samples. No significant differences (*p* ≥ 0.05) were found between these groups. Interestingly, only *C. difficile* was determined to have different abundances in CON animals under both scenarios (AB and NoAB). No significant differences in the relative abundance of species identified from unassembled reads were observed according to antibiotic exposure or AU/CON status, likely due to the high variability among samples.

MaAsLin2 analysis revealed a single species, *Streptomyces sp. REN17* to be higher in relative abundance in CON compared to AU samples (*p* < 0.01, q = 0.0200).

### Resistance genes and predicted metabolic activities

**Fig. 5:**
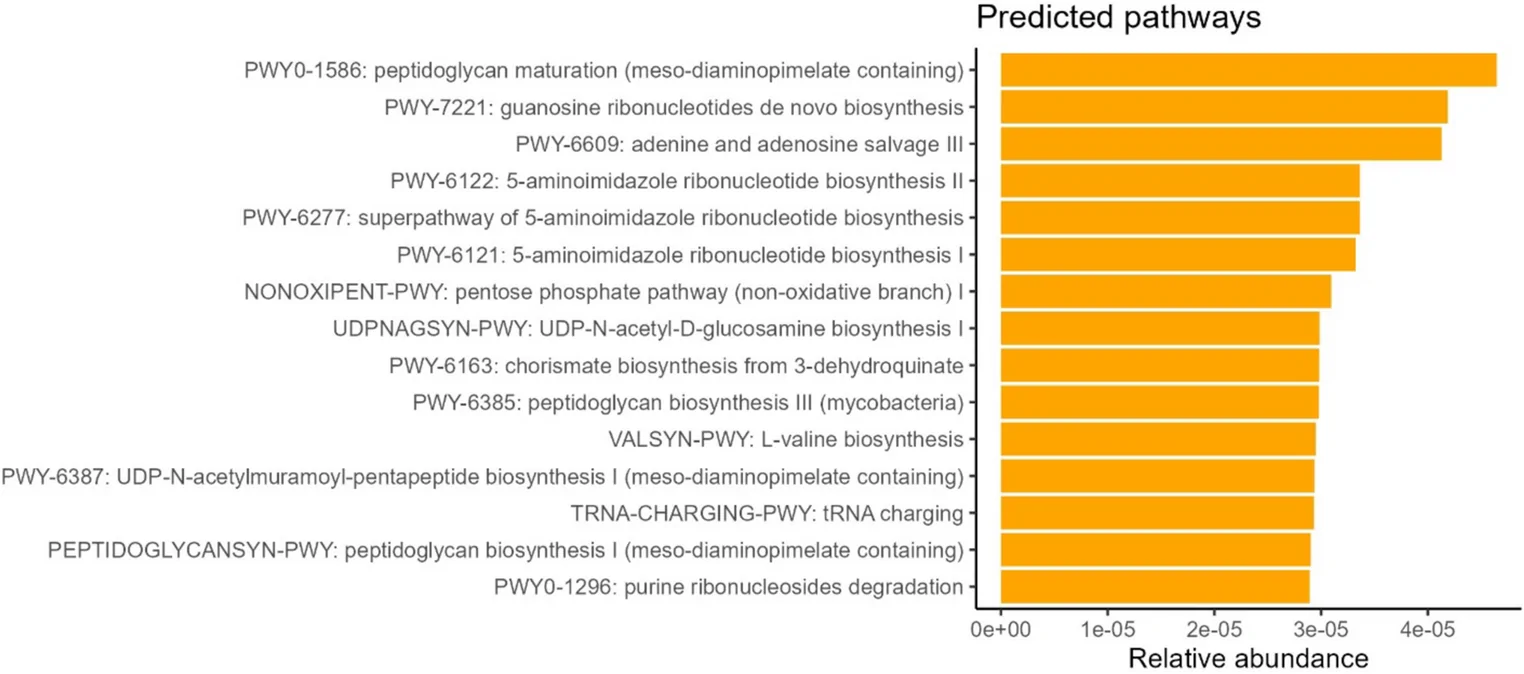
Most abundant predicted metabolic activities regardless of age or treatment as predicted by HUMAnN

Analysis of the unassembled reads using the AMRFinder+ database showed ARGs coding for phenicol macrolide (ribosome methylase), and tetracycline resistance in all calves regardless of tissue type or AB treatment (Supplementary Table 3). Analysis of predicted metabolic pathways was done with unassembled reads to assess possible variations between samples with AU or CON using the HUMAnN database. While there were differences in predicted activity (Fig. 5), none were considered significant.

## Discussion

The aim of this research was to assess the microbial diversity and composition, as well as the prevalence of antimicrobial resistance genes from western Canadian beef calves with and without AU. This type of research presents many challenges, including the use of heterogenous animals in the control group and the use of extended post-mortem intervals in the sampling scheme. While the latter can influence microbial community composition, all samples were analyzed for potential outliers based on 16S rRNA gene relative abundances at the phyla level. No detectable variation was observed in the later post-mortem sampling times and therefore, no samples were removed based on this factor.

Previous research has indicated that there is high probability that the gut microbiome has a role in the multifactorial etiology of AU (Hund and Wittek 2018). In the current study, no effect of tissue type (AU vs. CON) was seen in both the metataxonomic and the metagenomic analyses. However, due to a lack of high-quality bins created in the assembly of metagenomic sequences the taxonomic classification of sequences was only performed using unassembled reads and a higher resolution of microbiota could not be reached. The reduced quality may be explained in part as being due to the time of sampling postmortem (2–24 h) or the impact of long-term storage on the quality of material. The sequencing depth could have also impacted the outcome of the genomic assembly, due to high prevalence of host DNA and low bacterial load in the abomasum. Future studies should consider not only a shorter storage time, but also a maximum period for sampling postmortem, as well as an increased sequencing depth to improve the amount of reads to be used for contigs binning (Treichel et al. 2026).

In contrast to these results, previous culture-based studies showed *Clostridium perfringens* and *Campylobacter jejuni* being described as major bacterial agents in the AU of calves (Roeder et al. 1987; Mills et al. 1990). However, the relationship between bacterial presence and AU presence is still undetermined and culture dependent and independent studies have shown no difference in the presence of both *Clostridium* spp, and *C. perfringens* in the abomasum from healthy versus AU calves with low grade non-perforating ulcers (Valgaeren et al. 2013; Hund et al. 2015). While in the current study *Clostridium* was not found to be among the most prevalent taxonomy, it was found across all samples regardless of AU or CON. This would seem to indicate the presence of these bacteria as part of the commensal population of the abomasum with the opportunistic potential to become pathogenic under stress or dysbiosis (Mehdizadeh Gohari et al. 2021). The highest prevalence for taxonomy at the family level was of *Staphylococcaceae* across all samples, with the most abundant species in that family being *Staphylococcus aureus.* As *S. aureus* is one of the leading pathogens for deaths associated with antimicrobial resistance and the emergence of antimicrobial resistance strains, the metagenomic data was analyzed for the up-regulation of antimicrobial resistance genes in relation to AU. This study only found polymyxin resistance to be present with no difference between CON and AU calves. This is not unexpected as ARGs are often difficult to identify without enrichment, and the analysis done in this study was completed with unassembled reads instead of larger alignments. While relying only on assemblies could bias the dataset against microbes that cannot be assembled, due to rare or large genomes, accuracy from short reads is significantly less especially when performing predicted functional profiling. As such the ARG analysis yielded limited evidence for disease associated resistance patterns and future studies should consider enrichment analysis of high-quality samples to ensure an accurate coverage for this analysis.

Similar to *S. aureus*, the other predominant bacteria found in this study based on metagenomic analysis showed ubiquity across samples but are known pathogens, including *Clostridioides difficile*, *Paeniclostridium sordellii*, and *Fusobacterium necrophorum*, and *Klebsiella pneumoniae*. Of these, *P. sordellii*, *F. necrophorum* and *K. pneumoniae* all have known roles in bacteremia or septicemia but can be found in the GI tract, and many such bacteria are the primary colonizers of the alimentary tract of newborn ruminants, including *Escherichia* spp. and *Salmonella* spp. (Arshad et al. 2021; Bandyopadhyay et al. 2021; Bayne and Edmondson 2021). However, in the current data set there were not enough samples to separate animals under 1 week of age and therefore, it is difficult to determine which bacteria are associated with transient development of the commensal populations versus those which are potentially due to dysbiosis. Furthermore, the majority of published data in young calves is based on fecal analysis at various time points and cannot provide adequate information regarding the abomasum mucosal adherent population.

Perhaps most interestingly, *Streptomyces sp. REN17* was found to be a significantly more abundant in CON calves. *Streptomyces* species have been reported to produce a wide range of secondary metabolites that may benefit intestinal health, including exopolysaccharides and antibiotics (Cuozzo et al. 2023). In calves orally challenged with *E. coli* K99 and given antibiotic support therapy (intramuscular gentamicin 20 mL for 2 days), *Streptomyces* along with *Prevotella* were decreased compared to challenged calves given multispecies probiotics and control calves (Wu et al. 2022). While the findings of the current study suggest an association between *Streptomyces* abundance and a lack of AU, the functional significance of this observation remains unclear, and further work is needed to better understand the role of these microbes in the context of the abomasal ecosystem.

The current knowledge regarding the commensal population of the abomasum, as well as its role in regulating the health of the tissue in early life is minimal. In the current study no variation was found with relation to bacterial diversity or species richness, which would support the hypothesis that the microbiome of an AU is not associated with the presence of a single highly abundant pathogen. However, previous studies have also shown that calves that are reported as having an illness (neonatal diarrhea, pneumonia) tended to have lower bacterial diversity (Oikonomou et al. 2013). Since all calves in this study were sampled during postmortem, there is a potential that illnesses other than AU impacted the diversity of the abomasal microbiota.

Previous studies have divided rumen development into three phases: the non-rumination phase (from birth to week 3); the transition phase (from week 3 to 8); and the rumination phase (from week 8 onwards) (Yáñez-Ruiz et al. 2015). In the current study, the samples tended to fall into either the non-rumination phase or the transition phase, which corresponded with previous research showing that AU in range beef calves showed a peak incidence between 3 and 6 weeks (Tulleners and Hamilton 1980). Jelinski et al. (1996a) reported that the 85.6% of calves dying from AU were under two months of age. This high susceptibility for AU was believed to be due to the transition in diet. During the pre-weaning and transitional periods, anaerobes belonging to *Firmicutes* and *Bacteroidetes* become predominant through gradual colonization and stabilization of the GIT. *Bacteroides* in the gut play a vital role in development of immunological tolerance to commensal microbiota (Malmuthuge and Guan 2017). While healthy abomasal microbiome data is not available in the literature, in the rumen of calves, *Bacteroidetes* can increase in abundance after birth to a peak of 18–75% at 6 weeks of age, generally in opposition with the abundance of *Firmicutes*; after this age the ratio of *Bacteroidetes* to *Firmicutes* decreases (Rey et al. 2014). This pattern is consistent with the values seen in the current study, in which younger calves (≤ 30 days) tended to showing a higher percentage of *Bacteroidetes* compared to older calves (> 30 days). Additionally, AB calves appeared to be associated with a shift in this ratio, with a higher *Firmicutes:Bacteroidetes* ratio observed in both age groups; however, this finding should also be interpreted in light of the study design limitations, including small sample size and multiple sources of biological variation. Generally, the development of the gut microbiota in dairy animals is age dependent from birth to adulthood, and the dynamic changes in the resistome may be closely related to the gut microbiota in dairy calves (Liu et al. 2019). However, interpretation of the current results is limited due to the disproportional number of samples between the two identified age categories. As such, future studies should identify animals from multiple age categories to better separate the physiological development and any potential changes related to the presence of AU.

In conclusion, we were not able to identify a microbiota unique to AU nor could we associate prior antimicrobial therapy to a specific microbiota, regardless of age. While a larger sample size would likely improve statistical power, which is currently limited by several factors including heterogeneity, sampling times, and sequencings depths, all of which are necessary to better understand the role of the microbiome in AU etiology due to large individual animal variation. Future studies with a larger sample size would better resolve the role of the microbiome in AU etiology and in relation to antibiotic usage and resistance dynamics.

## Supplementary Information

Below is the link to the electronic supplementary material.

Supplementary Material 1

## Data Availability

The datasets used and/or analyzed during the current study are available from the corresponding author on reasonable request.
