# Supplementary material for: Observational study of the microbiome of perforated abomasal ulcers in unweaned beef calves in Canada
Source: Vet Res Commun. 2026 Sep 18;50(6):578. doi: 10.1007/s11259-026-11526-4 (PMC13586056; doi:10.1007/s11259-026-11526-4)
Supplement: Supplementary file 1 [file 11259_2026_11526_MOESM1_ESM.docx]

**Supplemental Table 1.** Frequency of samples per category for descriptive variables of age, antibiotic treatment and tissue type. Age is separated into 30 days of age or less, and over 30 days of age.

| Tissue Type | Age | Antibiotic Treatment | Sample Frequency |
| --- | --- | --- | --- |
| Control | >30 | no | 1 |
| Control | >30 | yes | 5 |
| Control | ≤30 | no | 5 |
| Control | ≤30 | yes | 2 |
| Ulcer | >30 | no | 13 |
| Ulcer | >30 | yes | 5 |
| Ulcer | ≤30 | no | 4 |
| Ulcer | ≤30 | yes | 4 |

**Supplementary Table 2.** Analysis using T-test and Wilcoxon to evaluate the impact of various factors on the alpha diversity of abomasum tissue.

| **Tissue Type** | **Control (CON)** | **Ulcer**  **(AU)** | **SEM** | **P-value** | **F-value** | **Type** | **Wilcoxon** |
| --- | --- | --- | --- | --- | --- | --- | --- |
| Observed ASV | 178.3 | 147.8 | 38.27 | 0.458 | 0.52 | 1.457 | 0.152 |
| Chao1 | 2.35 | 2.19 | 0.367 | 0.759 | 0.21 | 0.069 | 0.946 |
| Shannon | 2.89 | 2.51 | 0.246 | 0.268 | 0.05 | 1.240 | 0.222 |
| Simpsons | 0.824 | 0.777 | 0.0452 | 0.378 | 1.15 | 0.669 | 0.507 |
| **Age** | **≤30 days** | >30 **days** | **SEM** | **P-value** | **F-value** | **Type** | **Wilcoxon** |
| Observed ASV | 176.5 | 147.1 | 28.96 | 0.473 | 0.52 | 0.308 | 0.760 |
| Chao1 | 2.16 | 2.39 | 0.373 | 0.653 | 0.21 | -0.212 | 0.833 |
| Shannon | 2.57 | 2.65 | 0.242 | 0.818 | 0.05 | -0.529 | 0.599 |
| Simpsons | 0.744 | 0.811 | 0.0443 | 0.291 | 1.15 | -0.928 | 0.359 |
| **Antibiotic Usage** | **No** | **Yes** | **SEM** | **P-value** | **F-value** | **Type** | **Wilcoxon** |
| Observed ASV | 142.5 | 171.9 | 28.77 | 0.484 | 0.50 | 0.000 | 1.000 |
| Chao1 | 1.80 | 3.00 | 0.366 | 0.025 | 5.50 | 2.051 | 0.048 |
| Shannon | 2.51 | 2.77 | 0.260 | 0.469 | 0.54 | 0.754 | 0.455 |
| Simpsons | 0.766 | 0.806 | 0.0469 | 0.552 | 0.36 | 1.008 | 0.320 |
| **Sex** | **Female** | **Male** | **SEM** | **P-value** | **F-value** | **Type** | **Wilcoxon** |
| Observed ASV | 166.3 | 187.8 | 50.10 | 0.771 | 0.09 | -0.688 | 0.499 |
| Chao1 | 2.15 | 1.99 | 0.587 | 0.849 | 0.04 | 0.264 | 0.796 |
| Shannon | 2.70 | 2.97 | 0.366 | 0.600 | 0.28 | 1.050 | 0.306 |
| Simpsons | 0.816 | 0.770 | 0.0643 | 0.671 | 0.27 | 1.050 | 0.306 |

**Supplementary Table 3.** List of antimicrobial resistance (AMR), stress and virulence genes found within the unassembled read with AMRFinder+.

| **Gene symbol** | **Sequence name** | **Element type** | **Element subtype** | **Class** | **% Identity to reference sequence** | **Alignment length** | **Accession of closest sequence** |
| --- | --- | --- | --- | --- | --- | --- | --- |
| blaTEM-116 | broad-spectrum class A beta-lactamase TEM-116 | AMR | AMR | BETA-LACTAM | 100 | 286 | WP_000027050.1 |
| tet(44) | tetracycline resistance ribosomal protection protein Tet(44) | AMR | AMR | TETRACYCLINE | 98.08 | 364 | WP_063855884.1 |
| aph(6)-Id | aminoglycoside O-phosphotransferase APH(6)-Id | AMR | AMR | AMINOGLYCOSIDE | 100 | 278 | WP_000480968.1 |
| mdtM | multidrug efflux MFS transporter MdtM | AMR | AMR | EFFLUX | 96.68 | 211 | AAC77293.1 |
| fieF | CDF family cation-efflux transporter FieF | STRESS | METAL | NA | 100 | 300 | BAE77395.1 |
| acrF | multidrug efflux RND transporter permease subunit AcrF | AMR | AMR | EFFLUX | 99.47 | 569 | AAC76298.1 |
| emrD | multidrug efflux MFS transporter EmrD | AMR | AMR | EFFLUX | 100 | 394 | BAE77620.1 |
| tet(Q) | tetracycline resistance ribosomal protection protein Tet(Q) | AMR | AMR | TETRACYCLINE | 98.6 | 641 | WP_063856407.1 |
| tetA(P) | tetracycline efflux MFS transporter TetA(P) | AMR | AMR | TETRACYCLINE | 100 | 420 | WP_006440994.1 |
| tetB(P) | tetracycline resistance ribosomal protection protein TetB(P) | AMR | AMR | TETRACYCLINE | 96.43 | 336 | WP_012478245.1 |
| tet(O) | tetracycline resistance ribosomal protection protein Tet(O) | AMR | AMR | TETRACYCLINE | 99.52 | 624 | WP_050824634.1 |
| cfr(C) | 23S rRNA (adenine(2503)-C(8))-methyltransferase Cfr(C) | AMR | AMR | LINCOSAMIDE/MACROLIDE/STREPTOGRAMIN | 98.85 | 348 | WP_111690898.1 |
| espX1 | type III secretion system effector EspX1 | VIRULENCE | VIRULENCE | NA | 91.12 | 473 | ADD54679.1 |
| blaEC | BlaEC family class C beta-lactamase | AMR | AMR | BETA-LACTAM | 99.03 | 207 | AAC77110.1 |
| fdeC | intimin-like adhesin FdeC | VIRULENCE | VIRULENCE | NA | 96.05 | 1064 | ABE05822.1 |
| iss | increased serum survival lipoprotein Iss | VIRULENCE | VIRULENCE | NA | 100 | 82 | CAR12622.1 |
| lpfA | long polar fimbria major subunit LpfA-O113 | VIRULENCE | VIRULENCE | NA | 100 | 190 | AAT76975.1 |
| sul2 | sulfonamide-resistant dihydropteroate synthase Sul2 | AMR | AMR | SULFONAMIDE | 100 | 271 | WP_001043260.1 |
| arsC | glutaredoxin-dependent arsenate reductase | STRESS | METAL | ARSENIC | 93.62 | 141 | AAA21096.1 |
| asr | acid resistance repetitive basic protein Asr | STRESS | ACID | NA | 98.84 | 86 | BAA15331.2 |
| arsR | As(III)-sensing metalloregulatory transcriptional repressor ArsR | STRESS | METAL | ARSENIC | 100 | 117 | BAE77793.1 |
| tet(W) | tetracycline resistance ribosomal protection protein Tet(W) | AMR | AMR | TETRACYCLINE | 98.9 | 639 | WP_002586627.1 |
